# Treatment strategies for extremity sarcoma patients: a population-based analysis on German clinical cancer registry data

**DOI:** 10.3389/fonc.2025.1555502

**Published:** 2025-05-29

**Authors:** Jörg Andreas Müller, Karl-Stefan Delank, Kevin Laudner, Anne von Rüsten, Constanze Schneider, Jessica Isabel Selig, Ian Wittenberg, Alexander Zeh, Dirk Vordermark, Daniel Medenwald

**Affiliations:** ^1^ Department of Radiation Oncology, University Hospital Halle (Saale), Halle (Saale), Germany; ^2^ Department of Orthopedics, Trauma, and Reconstructive Surgery, University Hospital Halle (Saale), Halle (Saale), Germany; ^3^ Department of Health Sciences, University of Colorado Colorado Springs, Hybl Sports Medicine and Performance Center, Colorado Springs, CO, United States; ^4^ Clinical-epidemiological Cancer Registry Brandenburg-Berlin (Klinisch-epidemiologisches Krebsregister Brandenburg-Berlin gGmbH), Cottbus, Germany; ^5^ Cancer Registry Saxony, Dresden, Germany; ^6^ Clinical Cancer Registry Saxony-Anhalt (Klinische Krebsregister Sachsen-Anhalt GmbH), Magdeburg, Germany; ^7^ University Clinic for Radiation Therapy, University Hospital Magdeburg A. ö. R, Magdeburg, Germany

**Keywords:** extremity sarcoma, sarcoma, cancer registry research, German cancer registry data, treatment pattern, treatment outcomes

## Abstract

**Background:**

Sarcomas represent a heterogenous group of neoplasms, and there is a lack of data describing treatment patterns in Germany. The specific aim of this study was to evaluate treatment strategies and therapeutic outcomes of extremity sarcoma based on German cancer registry data.

**Methods:**

From 2000 to 2023, we identified n=3,094 patients diagnosed with extremity sarcoma from the German clinical cancer registries of Brandenburg-Berlin, Saxony and Saxony-Anhalt. Using logistic regression and Cox-proportional hazard analysis, we determined predictors of overall survival (OS). Propensity-score matching (PSM) was used to balance covariates and to reduce potential bias. We included sex, age at diagnosis, tumor localization, histological grade, Eastern Cooperative Oncology Group (ECOG) performance status, T-status and treatment as parameters in our regression models. To assess the robustness of our findings in the presence of missing data, we conducted a sensitivity analysis using multiple imputation.

**Results:**

A total of 2,240 propensity score-matched patients with extremity sarcomas were included. In multivariable Cox regression, higher age, high tumor grade, and advanced T-status were significantly associated with increased mortality. Treatment with radiotherapy (RT) alone was linked to worse survival (HR 1.82, 95% CI 1.12–2.95, *p* = 0.015), whereas neoadjuvant RT and surgery alone showed no survival advantage compared to adjuvant RT. The median OS was longest for patients treated with surgery alone (194 months) and adjuvant RT (146 months), and shortest with RT alone (82 months). Sensitivity analyses using multiple imputation confirmed the robustness of the results.

**Conclusions:**

Adjuvant RT and surgery alone were associated with the most favorable survival outcomes in patients with extremity sarcomas. Advanced age, tumor grade, and T-stage were strong negative prognostic factors. RT without surgery was linked to significantly reduced survival.

## Introduction

Sarcomas represent a heterogenous group of neoplasms. They can broadly be categorized into:

- Sarcomas of soft tissues (including fat, muscle, nerve and nerve sheath, blood vessels, and other connective tissues).
- Sarcomas of bone (1).

Prior exposure to radiation therapy (RT) in the affected region is a known risk factor for the development of soft tissue sarcomas (STS) (2–4). Additional risk factors include exposure to certain chemicals—such as herbicides like Agent Orange—and underlying genetic syndromes, including Li-Fraumeni syndrome and neurofibromatosis (5).

To date, more than 50 distinct histologic subtypes of STS have been identified. While pulmonary metastases are the most common pattern of distant spread, intra-abdominal tumors tend to metastasize to the liver and peritoneum. The anatomic location of the primary tumor is a key factor influencing both treatment decisions and clinical outcomes. The most common primary sites for soft tissue sarcomas are the extremities (43%), followed by the viscera (19%), retroperitoneum (15%), trunk (10%), and head and neck region (9%) (6). Given the heterogeneity of STS with respect to both histological subtype and tumor location, this study focused specifically on extremity sarcomas (ETS), the most common primary site, accounting for 43% of all cases. This restriction allows for a more homogeneous analysis of treatment patterns and survival outcomes.

There are various therapeutic approaches for treating ETS. The primary treatment in early-stage sarcoma patients consists of a wide surgical resection to obtain tumor-free resection margins (7, 8). Positive surgical margins are a strong predictor of local recurrence (LR) for patients with extremity STS (9–14). In a large cohort study that examined the clinical significance of main predictors of LR in patients STS of extremity or trunk, the 10-year cumulative possibility of LR was significantly higher among patients with positive margins (23.9% vs. 9.2% for those with negative margins; p<0.001) (13). Amputation once was considered as standard treatment for ETS patients to achieve local control. However, Rosenberg et al. showed in a randomized control study of 43 patients that limb-sparing surgery with RT was an effective treatment in patients with high-grade STS of the extremities, with a LR rate of 15% and no difference in overall survival (OS) and disease-free survival (DFS) compared to amputation (15).

The use of preoperative or postoperative RT in appropriately selected patients is supported by several randomized studies (16–18) and retrospective analyses (19–23). Gingrich et al. investigated the impact of neoadjuvant RT and adjuvant RT on R0 resection rates and OS in ETS patients undergoing surgery, based on data from the American National Cancer Database. Their data showed that preoperative RT and postoperative RT was associated with increased OS. Additionally, preoperative RT was predictive of achieving R0 resection (23). Furthermore, similar local control and progression-free survival (PFS) rates were described in sarcoma patients receiving either preoperative or postoperative RT with localized primary or recurrent disease (18, 24). Treatment-related side effects were more common in patients treated with postoperative RT, which may be related to the higher RT dose (66 Gy vs. 50 Gy neoadjuvant RT) and the larger treatment volume (18, 25).

A prospective randomized study including 91 patients with high-grade lesions and 51 patients with low-grade lesions demonstrated the efficacy of postoperative RT following limb-sparing surgery (17, 26). The 10-year local recurrence (LR) rate was significantly reduced by postoperative RT among patients with high-grade lesions (no LRs in patients treated with surgery + RT vs. 22% in those who underwent surgery alone; p=0.0028). Moreover, outcomes at 20-year follow-up showed no benefit in OS for patients treated with adjuvant RT (26).

As predictors of time to LR radiotherapy, tumor side and resection margin status were found. According to the findings of Yang and Beane et al., no difference in OS was observed (27).

Preoperative chemoradiation therapy has been shown to improve the prognosis of OS, disease-free survival (DFS) and local control rates in patients with stage II-III sarcoma located on the extremity and trunk region. However, side effects such as acute reactions need to be considered under this therapeutic approach (28, 29).

We aim to evaluate patterns of care of ETS in a German federal state based on cancer registry data. However, there is a lack of studies investigating treatment patterns and clinical outcomes in STS, particularly in the context of real-world data. In Germany, in particular, data describing contemporary treatment strategies and their association with survival outcomes remain scarce. Such real-world insights are essential for clinicians, as they reflect current clinical practice and can support evidence-based decision-making in the management of this rare and heterogeneous disease. Our findings will be compared to the guideline recommendations and prospective multicentric trials.

In this study, we hypothesized that postoperative or preoperative radiotherapy would be associated with superior OS compared to surgery alone in patients with ETS, based on real-world registry data. In addition, we aimed to evaluate the prognostic impact of established clinical factors such as performance status, tumor size, and histological grade on survival outcomes.

## Methods

### Data and material

For our study, data from the clinical cancer registries of Saxony, Saxony-Anhalt and Brandenburg-Berlin were analyzed. These population-based registries are regulated by German federal and state law and incorporates data that are transferred from healthcare facilities in Saxony-Anhalt and Berlin-Brandenburg.

Among other information, data sets included structured information on tumor, node, metastasis (TNM)-stage, grading and histology, date of birth, cause and date of death, date of diagnosis (month as smallest temporal unit in each date variable) and treatment. Furthermore, information on treatment procedures such as administered radiation dose and the timepoint of the first and last radiation treatment or number of surgeries were included. Additionally, the TNM-stage referred to in this data set, included the clinical or pathological stage (if an operation was performed). In order to estimate clinical treatment strategies, clinical stages were used if both ratings differed. Some cases showed incomplete information. If information on subclassification of T-stages (e.g., T1a, T1b) were not available, cases were classified as subgroups T1-4. Likewise, Union for International Cancer Control (UICC)-stages were defined as I-IV. Age groups were defined as follows: <50 years, 51–60 years, 61–70 years, 71–80 years and older than 80 years.

The clinical performance status was documented according to the Eastern Cooperative Oncology Group (ECOG). Fully active patients were classified as grade 0 by the study group investigators. Patients restricted in physically activity but ambulatory were classified as grade 1. Higher grades describe disabled patients, which were unable to carry out work activities and limited in selfcare. Grade 5 was used for deceased patients (30). Information on the ECOG performance status were only available for the registry data of Brandenburg-Berlin and Saxony, which may have led to a cancer registry-related bias.

Because of the large number of histological subtypes, the cut-off for inclusion in the study was set at greater than five documented cases for each histological subgroup. Subtypes meeting this threshold were reported individually in the analysis. STS cases of the extremity that did not meet this threshold or were not assigned to a more specific histological category were grouped under the label “unclassified soft tissue sarcoma” for analytical purposes. Although all included cases were classified as STS by definition, this residual category referred specifically to those STS cases for which no further histological specification beyond general STS coding was available, or which were too rare to be meaningfully analyzed as separate subgroups. The remaining rare or unspecified histologies were summarized under “other histological subtype.” A detailed breakdown of all histological subgroups is provided in the supplement (**Supplementary Table S1**).Tumor localization was defined based on International Statistical Classification of Diseases and Related Health Problems (ICD)-codes for sarcoma cases. All ICD-codes C49.0-C49.9 were used to assign tumor locations for all recorded sarcoma cases. We did not define tumor locations for organ related sarcomas because of differing ICD-codes and classification criteria (e.g., Pleural Mesothelioma, Mesothelioma, Gastrointestinal stromal tumors). Furthermore, tumor size was estimated by T-stage in accordance with the American Joint Committee on Cancer (AJCC) staging criteria (Cates 2018). Histological grades were defined as low and high grade sarcoma according to the ‘Arbeitsgemeinschaft Deutscher Tumorzentren, ADT’ (31). G1 tumors were classified as low-grade sarcomas, while all tumors with a grading of ≥ G2 were categorized as high-grade sarcomas.

### Treatment assignment and therapy-related data

To assess how patients were selected for different treatment modalities, therapy-related data from the cancer registries were analyzed in detail. Each registry provided OPS (Operationen- und Prozedurenschlüssel) codes with corresponding dates for up to 17 distinct surgical procedures per patient, including documentation of treatment intent (curative, palliative, or diagnostic) and information on potential perioperative complications. Additionally, data on the resection status (R-status) and the localization of residual tumor were included. The number of examined and affected lymph node stations was also recorded, offering further insight into surgical extent and staging.

For radiotherapy, data were provided on up to 13 partial radiation treatments per patient, including details on irradiation technique, application modality, and physical units of applied dose. For each partial treatment, both single fraction doses and total dose could be determined. Importantly, the data indicated whether radiotherapy had to be terminated prematurely, and whether concurrent chemotherapy was administered. Furthermore, treatment toxicity was documented, where available. Radiotherapy target volumes were classified as treatment of the primary tumor, lymph nodes, metastases, or other anatomical sites, and treatment intent (curative, palliative) was recorded.

Where available, the variable *“Stellung der Therapie im Konzept”* (“position of the therapy in the treatment concept”) allowed classification of radiotherapy as preoperative or postoperative. Due to a high proportion of missing values in this variable (52%), the classification of radiotherapy as preoperative or postoperative was primarily based on the chronological sequence of curatively intended surgery and radiotherapy start dates. Postoperative RT was defined as RT starting after surgery with a curative intent and a total dose of ≥40 Gy to distinguish it from palliative RT. Conversely, RT was classified as preoperative if it preceded surgery with curative intent. Cases with missing or inconsistent date patterns or total doses <40 Gy were excluded from comparative analysis of RT sequencing.

We assessed the resection-status based on all curative intent surgeries. The resection status was considered R0 even if an R0-status was only achieved after multiple resections. RX-resected patients were labeled separately from patients without any available information regarding the resection status.

### Definition of periods

Cases diagnosed between 2000 and 2023 (most recent data with sufficient quality) were included for this analysis. Cases were censored in January 2023 (latest complete recording of death) or after 60 months to avoid a bias due to cases that died in more recent years, but whose changed survival status had not yet been considered in the data.

For the cancer registry of Brandenburg-Berlin a date of 31.12.2021 was set as censoring date for survival analysis.

### Statistical analyses

We used proportional hazard Cox-regression models to assess the association of cancer-related parameters with mortality and computed hazard ratios (HR) with 95% confidence intervals (CI).

To reduce the immortal time bias, survival times were defined based on the time interval between the end of the last treatment and death. In order to reduce potential selection bias, we used propensity score matching and adjusted our model for age, sex, histological grade, T-stage and ECOG performance status.

Furthermore, we computed univariate Cox-regression models. As potential prognostic factors histological grading (as defined above), age at diagnosis, T-stage, M-stage, tumor localization based on ICD-codes as described above, ECOG performance status, treatment and patient sex were included in our regression models. For illustrative purposes, Kaplan-Meier curves were created for some included risk factors. Our primary endpoint was overall survival (OS). Additionally, we computed median OS survival rates.

To assess the robustness of our findings in the presence of missing data, we conducted a sensitivity analysis using multiple imputations. We selected a subset of relevant variables for imputation, including survival time (survived months), event indicator (death), and covariates of clinical interest.

Multiple imputation was performed using the *mice* package in R (version 3.14.0), employing predictive mean matching with five imputed datasets and a random seed for reproducibility.

For each imputed dataset, we fitted a Cox proportional hazards model. Covariates included in the model were selected based on clinical relevance and prior evidence. The results from the five imputed datasets were pooled using Rubin’s rules to obtain combined estimates and standard errors.

This approach allowed us to account for uncertainty due to missing data and evaluate whether the main conclusions were consistent across imputed datasets.

A significance level of 0.05 was used. All statistical analyses were performed using RStudio Version 1.4.1717 (RStudio 2020, Integrated Development for R. RStudio, PBC, Boston, MA, USA). All graphics were computed with the “gtsummary” package in RStudio (32).

## Results

### Case selection

**Figure 1:**
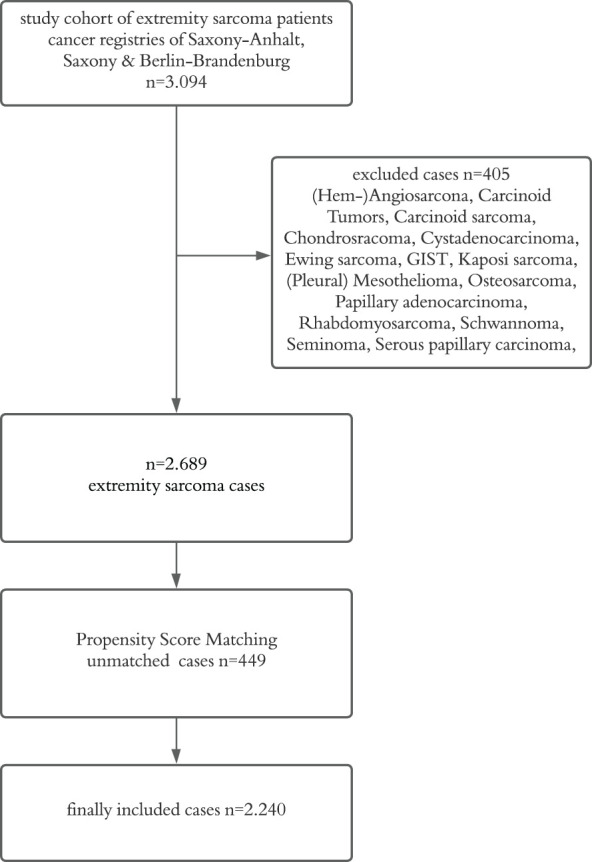
Flowchart of sample inclusion and exclusion criteria (GIST, gastrointestinal stromal tumor).

The total number of extremity sarcoma cases diagnosed between 2000 and 2023, provided by the cancer registries of Saxony-Anhalt, Saxony and Berlin-Brandenburg was n=3,094 (cases were reported in accordance with the German manual of cancer registration (31)). In order to meet the specific aim of this study, we excluded subgroups with different tumor biology and low number of cases. Consequently, all patients diagnosed with (hem)angiosarcoma, carcinoid tumors, carcinoid sarcoma, chondrosarcoma, cystadenocarcinoma, Ewing sarcoma, gastrointestinal stromal tumors (GIST), Kaposi sarcoma, (pleural) mesothelioma, osteosarcoma, papillary adenocarcinoma, rhabdomyosarcoma, schwannoma, seminoma and serous papillary carcinoma were excluded from further analysis. Incomplete data with undefined histological subtypes or lacking ICD-codes were removed from the study, leading to a total number of n=405 excluded cases. Propensity score matching (PSM) was performed afterwards. This resulted in a total number of 2,240 propensity score matched cases included for analysis (**Figure 1**).

### Patient characteristics

Patient characteristics are summarized in **Table 1**. Most patients were male (56%) with a median age of 64 years (IQR 51–76). The most common histological subtypes were soft tissue sarcoma NOS (24%), liposarcoma (21%), leiomyosarcoma (14%), and pleomorphic sarcoma (11%). High-grade tumors were present in 44%, while 21% lacked grading information.

**Table 1:** Patient characteristics of all propensity score matched patients.

Characteristic	N = 2,240*^1^*
Sex	
Male	1,265 (56%)
Female	975 (44%)
Age (years)	64 (51, 76)
Histological subtype	
Clear Cell Sarcoma	20 (0.9%)
Dermatofibrosarcoma Protuberans	16 (0.7%)
Epithelioid Sarcoma	24 (1.1%)
Fibromyxoid Sarcoma	44 (2.0%)
Fibrosarcoma	59 (2.6%)
Leiomyosarcoma	322 (14%)
Liposarcoma	466 (21%)
Malignant Fibrous Histiocytoma	32 (1.4%)
Malignant Peripheral Nerve Sheath Tumor	19 (0.8%)
Malignant Solitary Fibrous Tumor	2 (<0.1%)
Myxofibrosarcoma	215 (9.6%)
Myxosarcoma	18 (0.8%)
Pleomorphic Sarcoma	238 (11%)
Unclassified Soft Tissue Sarcoma	536 (24%)
Spindle Cell Sarcoma	83 (3.7%)
Synovial Sarcoma	90 (4.0%)
Undifferentiated Sarcoma	56 (2.5%)
Histological grade	
High grade	978 (44%)
Low grade	794 (35%)
Unknown	468 (21%)
T-status	
1	386 (17%)
2	947 (42%)
3	136 (6.1%)
4	160 (7.1%)
Unknown	492 (22%)
X	119 (5.3%)
N-status	
0	1,429 (64%)
1	45 (2.0%)
Unknown	544 (24%)
X	222 (9.9%)
M-status	
0	1,470 (66%)
1	185 (8.3%)
Unknown	534 (24%)
X	51 (2.3%)
ECOG-status	
0	211 (9.4%)
1	158 (7.1%)
2	60 (2.7%)
3	13 (0.6%)
4	4 (0.2%)
Unknown	1,794 (80%)
Treatment	
Adjuvant RT	749 (33%)
Neoadjuvant RT	141 (6.3%)
RT alone	169 (7.5%)
Surgery alone	122 (5.4%)
Systemic Therapy + RT	2 (<0.1%)
Systemic Therapy alone	96 (4.3%)
Unknown	961 (43%)
Tumor localization	
Lower Extremity	1,697 (76%)
Upper Extremity	543 (24%)
Tumor side	
Both Sides	1 (<0.1%)
Left Side	726 (32%)
Midline	1 (<0.1%)
Right Side	729 (33%)
Unknown	783 (35%)
Tumor size	
<=5cm	386 (17%)
>10cm and <15cm	136 (6.1%)
>15cm	160 (7.1%)
>5cm and <10cm	947 (42%)
Unknown	611 (27%)

*^1^* n (%); Median (IQR).

The majority of tumors were T2 (42%) or T1 (17%), with T3/T4 stages accounting for 13%. T-stage was unknown in 22%. Most patients were N0 (64%) and M0 (66%); nodal and distant metastases were uncommon (2% and 8%, respectively). ECOG performance status was missing in 80% of cases; among documented cases, most had ECOG 0–2.

Adjuvant RT was the most frequent treatment (33%), followed by neoadjuvant RT (6.3%) and RT alone (7.5%). Surgery without additional therapy was used in 5.4%, and systemic therapy alone in 4.3%. Treatment data were unavailable for 43% of patients.

Tumors were predominantly located in the lower (76%) or upper (24%) extremities. Tumor laterality was balanced between left (32%) and right (33%) sides, though 35% were undocumented. Tumor size was most often 5–10 cm (42%); 27% lacked size documentation.

**Table 2** summarizes the resection status of all patients who underwent surgery (n = 1,012).

**Table 2:** Resection status of patients treated with surgery.

Characteristic	Overall, N = 1,012*^1^*	Adjuvant RT, N = 749*^1^*	Neoadjuvant RT, N = 141*^1^*	Surgery alone, N = 122*^1^*	P-value*^2^*
Resection status					0.11
R0-Status	566 (77%)	466 (75%)	100 (85%)	0	
R1-Status	105 (14%)	95 (15%)	10 (8.5%)	0	
R2-Status	10 (1.4%)	8 (1.3%)	2 (1.7%)	0	
RX-Status	55 (7.5%)	49 (7.9%)	6 (5.1%)	0	
Unknown	276	131	23	122	

*^1^* n (%).

*^2^* Fisher’s exact test.

The majority of patients achieved an R0 resection (77%), indicating complete tumor removal, while 14% had microscopic (R1) and 1.4% macroscopic (R2) residual disease. In 7.5% of cases, the resection status was indeterminate (RX).

Among those treated with neoadjuvant radiotherapy, 85% achieved R0 status, compared to 75% in the adjuvant RT group. The resection status was unknown in 276 patients.

### Propensity score matching

We used Propensity score analyses to adjust our models to reduce selection bias. All included cases were matched with one control case based on each measured propensity score. We used the Nearest Neighbor method to perform our analyses. Overall, a sample size of n=2,689 cases was included. 2,240 cases could be matched in the control and treatment group. Radiation therapy was defined as treatment variable, any treatment without RT was stated as control variable.

In short, the treated and control cases after matching were very similar with respect to of age, ECOG performance status, sex, histological grade and TNM classification (**Supplementary Table S1** of the supplement shows the numeric results of propensity score matching).

**Figure 2:**
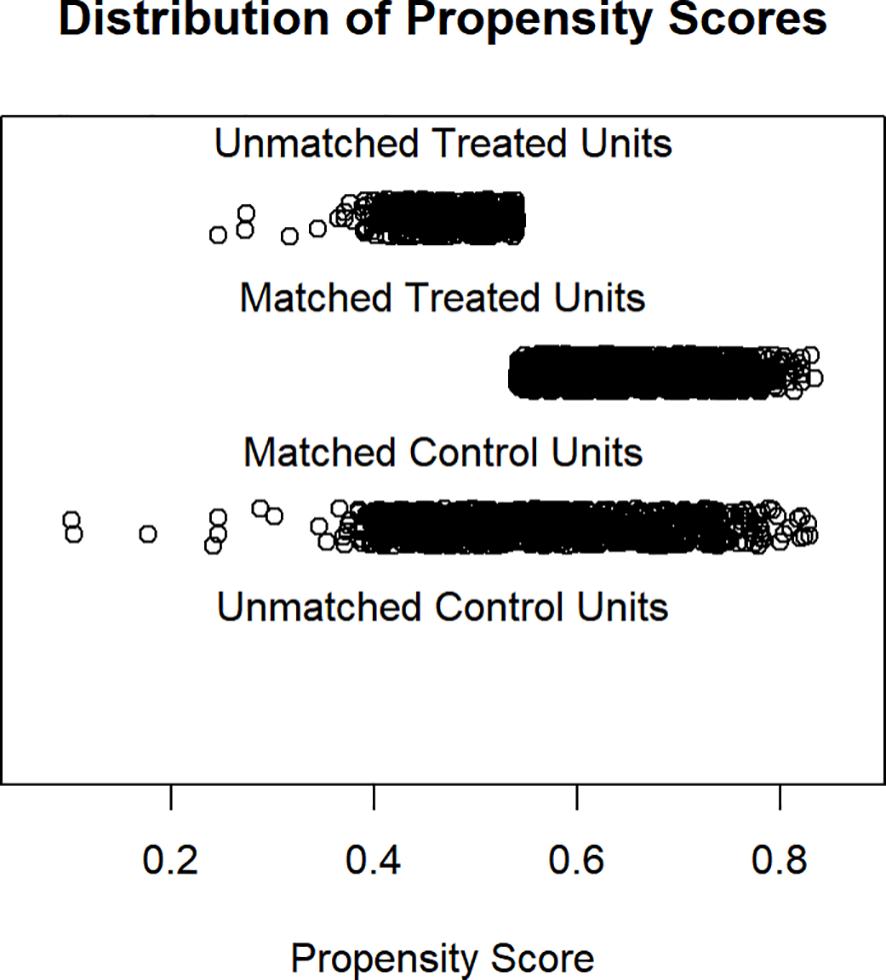
Distribution of propensity scores.

**Figure 2** is a jitter plot where each case represents a case`s propensity score. The absence of cases in the uppermost stratification indicates that there were no unmatched treatment units. The middle stratifications show the close match between the treatment units and the matched control units. The final stratification shows the unmatched control units, which was not be used in any further analyses.

**Figure 3:**
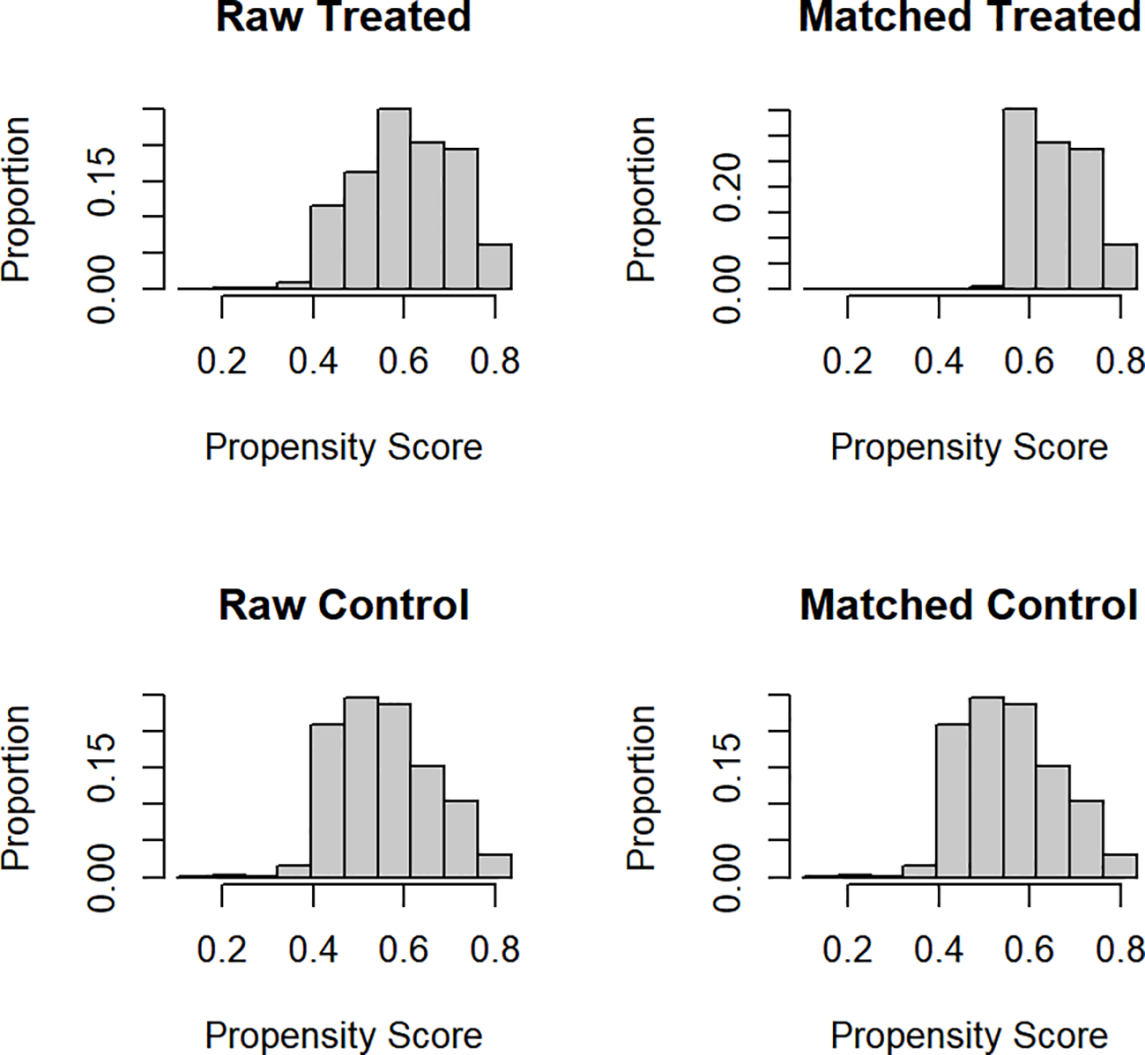
Histograms of propensity scores before and after matching.

**Figure 3** shows the histograms before and after matching. The histograms before matching on the left differ to a great degree. The histograms after matching on the right are very similar somehow. In summation, both the numerical and visual data show that the matching was successful.

### Survival analyses

Univariate and multivariate Cox regression analyses for OS are summarized in **Table 3**.

**Table 3:** Univariate and multivariate Cox-regression models based on OS.

Characteristic	Univariable Analyse				Multivariable Analyse		
	N	HR*^1^*	95% CI*^1^*	P-value	HR*^1^*	95% CI*^1^*	P-value
Sex	2,240			<0.001			
Male		—	—		—	—	
Female		0.79	0.70, 0.91		0.76	0.56, 1.04	0.083
Age group	1,097			<0.001			
<50		—	—		—	—	
>80		5.27	3.75, 7.42		5.70	3.08, 10.6	<0.001
51-60		1.26	0.85, 1.87		1.59	0.81, 3.13	0.2
61-70		1.97	1.42, 2.72		2.34	1.36, 4.03	0.002
71-80		2.74	1.98, 3.79		3.48	1.94, 6.21	<0.001
Tumor localization	2,240			0.050			
Lower Extremity		—	—				
Upper Extremity		0.86	0.73, 1.00				
Histological grade	2,240			<0.001			
High Grade		—	—		—	—	
Low Grade		0.53	0.46, 0.62		0.63	0.41, 0.97	0.034
Unknown		0.52	0.44, 0.62		0.55	0.36, 0.82	0.004
T status	2,240			<0.001			
1		—	—		—	—	
2		1.42	1.11, 1.80		0.75	0.46, 1.24	0.3
3		8.42	5.85, 12.1		4.78	1.81, 12.6	0.002
4		8.62	6.13, 12.1		5.89	2.69, 12.9	<0.001
Unknown		1.15	0.89, 1.49		0.65	0.38, 1.13	0.13
X		1.74	1.23, 2.46		1.24	0.61, 2.54	0.5
Treatment	1,181			<0.001			
Adjuvant RT		—	—		—	—	
Neoadjuvant RT		1.89	1.40, 2.54		1.04	0.49, 2.23	>0.9
RT alone		2.47	2.00, 3.05		1.82	1.12, 2.95	0.015
Surgery alone		0.68	0.48, 0.96		1.04	0.66, 1.64	0.9
ECOG status	2,240			0.003			
>1		—	—		—	—	
0		0.59	0.41, 0.85		1.11	0.36, 3.44	0.9
1		0.64	0.46, 0.90		0.83	0.38, 1.82	0.6
Unknown		0.84	0.64, 1.10		1.20	0.59, 2.45	0.6

*^1^* HR, Hazard Ratio; CI, Confidence Interval.

In the univariate analysis, female patients had a significantly better survival compared to males (HR 0.79, 95% CI 0.70–0.91). However, this difference was not statistically significant in the multivariate model (HR 0.76, 95% CI 0.56–1.04, p = 0.083).

Age was strongly associated with survival. Compared to patients under 50 years, those aged 71–80 (HR 2.74, 95% CI 1.98–3.79) and over 80 years (HR 5.27, 95% CI 3.75–7.42) had markedly higher mortality risks in the univariate analysis. This association persisted in the multivariate model, with patients over 80 showing the highest risk (HR 5.70, 95% CI 3.08–10.6, p < 0.001).

Tumor localization showed no significant association with survival in either model, although upper extremity tumors were associated with a slightly reduced risk in the univariate model (HR 0.86, 95% CI 0.73–1.00, p = 0.050).

Histological grade was significantly associated with OS. Low-grade sarcomas had a lower risk of death compared to high-grade sarcomas both in the univariate (HR 0.53, 95% CI 0.46–0.62) and multivariate analyses (HR 0.63, 95% CI 0.41–0.97, p = 0.034).

T-status showed a strong association with survival. Compared to T1 tumors, T3 (HR 8.42, 95% CI 5.85–12.1) and T4 (HR 8.62, 95% CI 6.13–12.1) were associated with significantly worse OS in the univariate analysis. These associations remained robust in the multivariate model (T3 vs. T1: HR 4.78, 95% CI 1.81–12.6, p = 0.002; T4 vs. T1: HR 5.89, 95% CI 2.69–12.9, p < 0.001).

Regarding performance status, patients with ECOG >1 had worse survival in the univariate analysis. Compared to these, patients with ECOG 0 (HR 0.59, 95% CI 0.41–0.85) and ECOG 1 (HR 0.64, 95% CI 0.46–0.90) had significantly better outcomes. These associations were not statistically significant in the multivariate analysis (ECOG 0: HR 1.11, 95% CI 0.36–3.44; ECOG 1: HR 0.83, 95% CI 0.38–1.82).

Treatment strategy was also associated with OS. In the univariate model, neoadjuvant RT (HR 1.89, 95% CI 1.40–2.54) and RT alone (HR 2.47, 95% CI 2.00–3.05) were associated with worse survival compared to adjuvant RT. Surgery alone was associated with improved survival (HR 0.68, 95% CI 0.48–0.96). In the multivariate model, only RT alone remained significantly associated with worse survival (HR 1.82, 95% CI 1.12–2.95, p = 0.015), while other treatment comparisons did not show significant differences.

**Figure 4:**
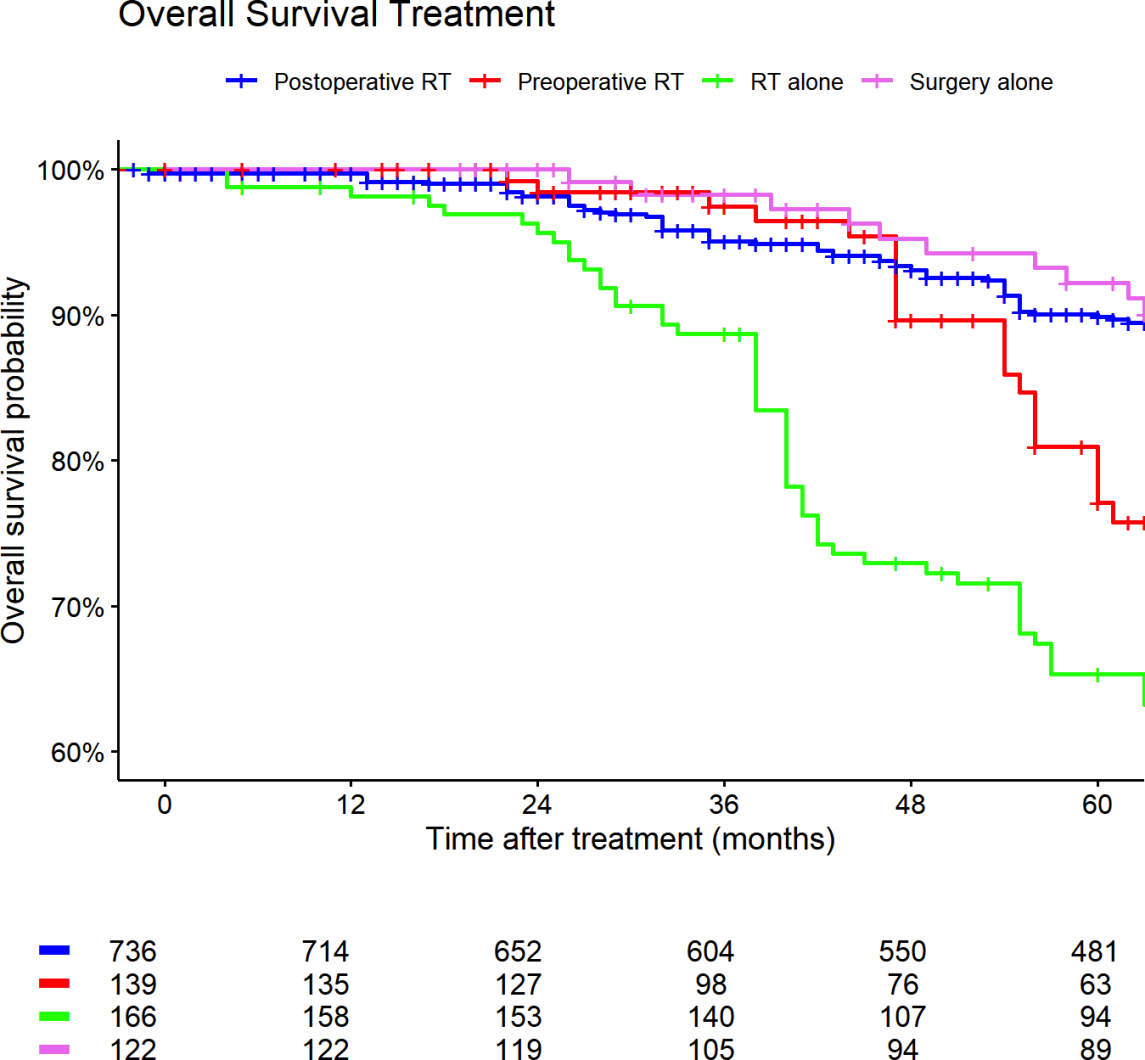
Kaplan-Meier curves of all patients (outcome: overall survival). The graph is based on treatment strategies. All graphs were censored 60 months after treatment. RT, radiotherapy.

Based on our regression model, we computed Kaplan-Meier curves for illustrative purposes. **Figure 4** shows the survival probabilities of all included PSM patients regarding treatment strategies. The corresponding median survival rates are illustrated in **Table 4**. In this cohort of patients with extremity sarcomas, the 3-year overall survival rate was 94.2%, and the 5-year overall survival rate was 86.4%. Median overall survival varied significantly between treatment groups (p < 0.001, log-rank test). Patients treated with adjuvant radiotherapy had the longest median survival of 146 months (95% CI 140–153). Those treated with surgery alone had a median survival of 194 months (95% CI 161–215), followed by neoadjuvant RT with 103 months (95% CI 98–144). The poorest outcome was observed in patients receiving radiotherapy alone, with a median overall survival of 82 months (95% CI 70–91).

**Table 4:** Median survival rates (months) based on treatment strategies. CI, confidence interval.

Characteristic	N	Median Overall survival (95% CI)	P-value*^1^*
Treatment	1,181		<0.001
Adjuvant RT		146 (140, 153)	
Neoadjuvant RT		103 (98, 144)	
RT alone		82 (70, 91)	
Surgery alone		194 (161, 215)	

*^1^* Log-rank test.

**Figure 5:**
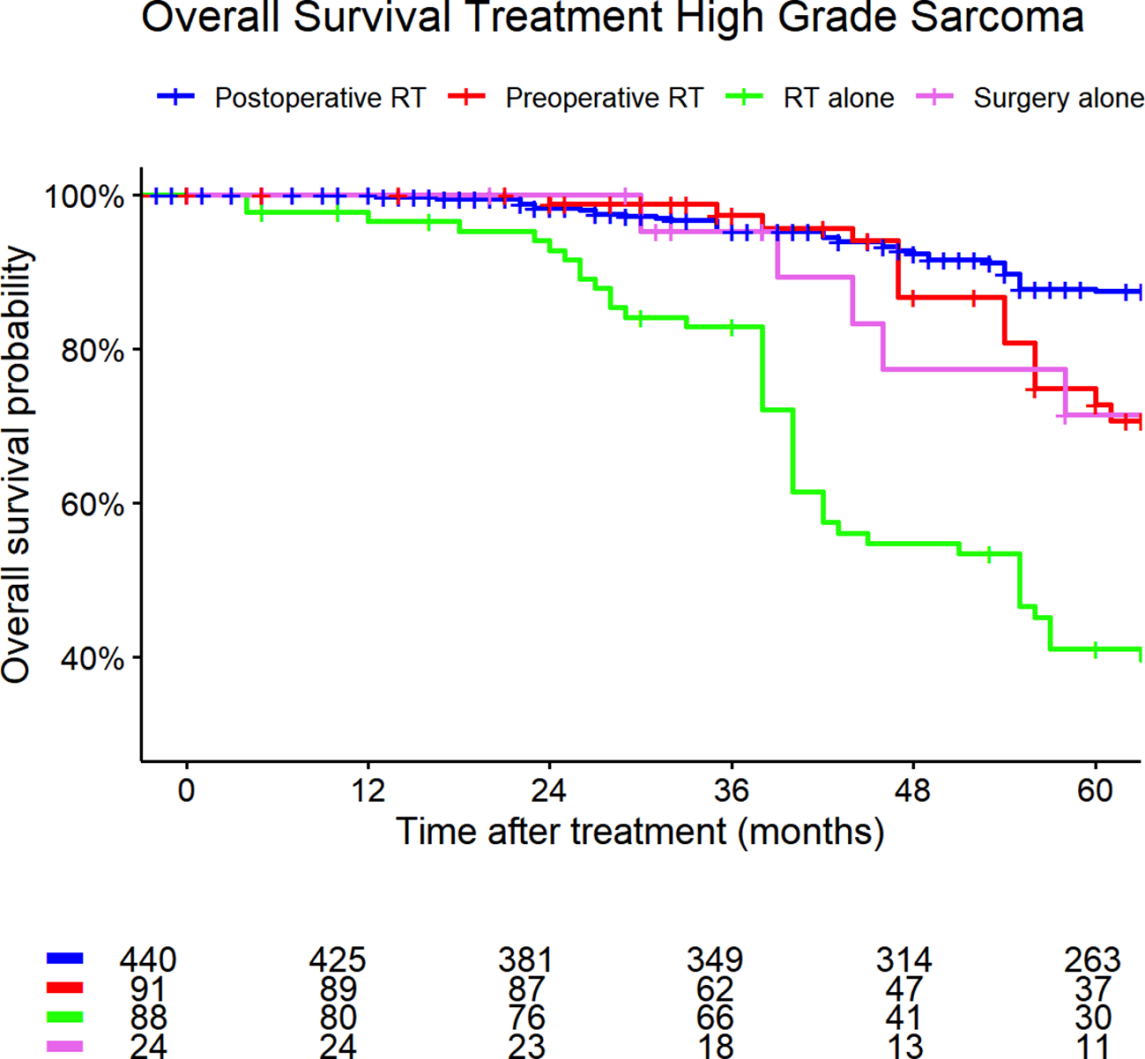
High-grade sarcoma. Kaplan-Meier curves of high- and low-grade sarcoma patients (outcome: overall survival). The graph is based on treatment strategies. All graphs were censored 60 months after treatment. RT, radiotherapy.

**Figure 6:**
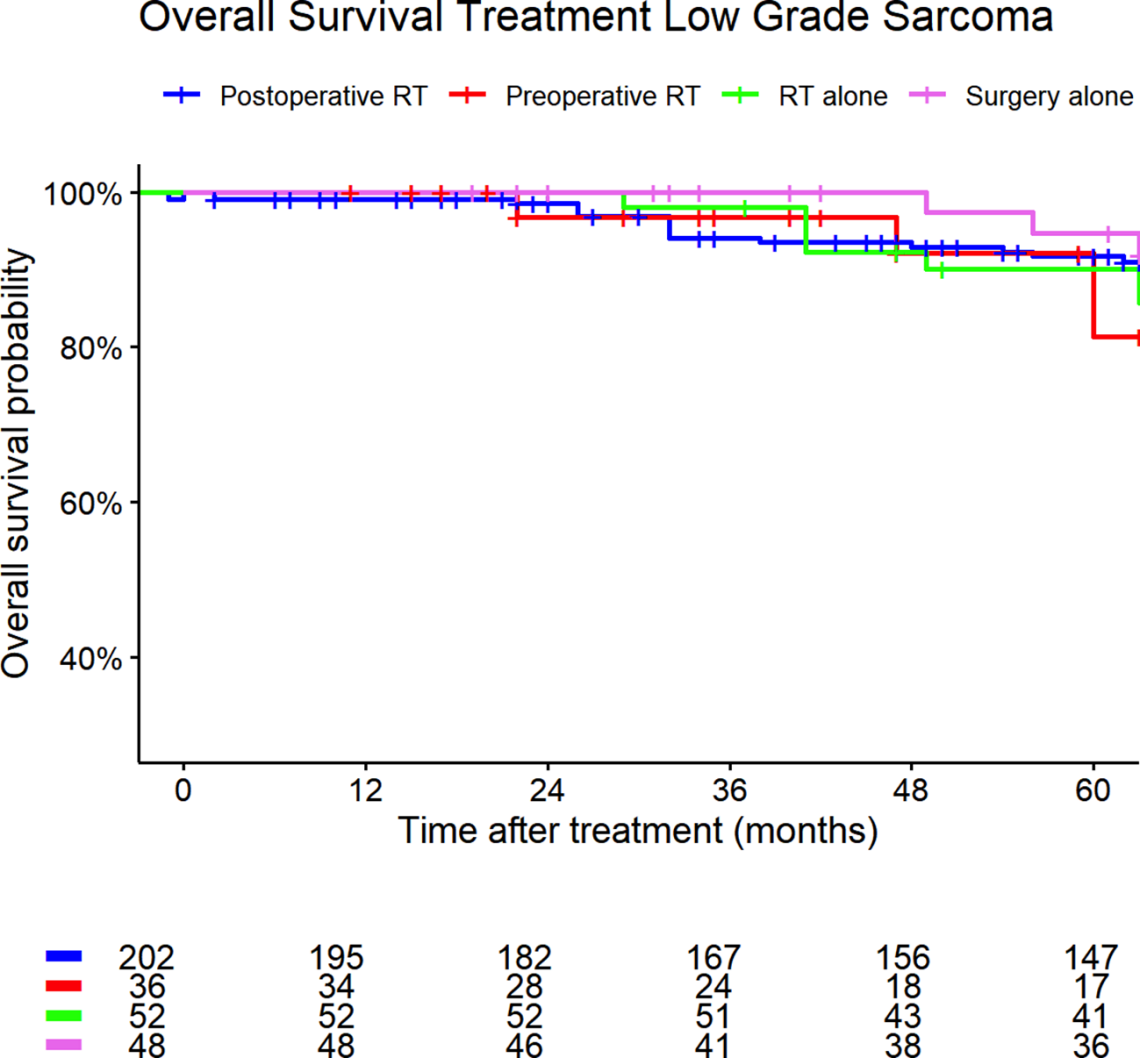
Low-grade sarcoma. Kaplan-Meier curves of high- and low-grade sarcoma patients (outcome: overall survival). The graph is based on treatment strategies. All graphs were censored 60 months after treatment. RT, radiotherapy.

**Figures 5**, **6** show the Kaplan-Meier curves of high- and low-grade sarcoma cases regarding different treatment strategies. The median overall survival was 119 months (95% CI: 108–128) for patients with high-grade sarcomas and 154 months (95% CI: 147–184) for those with low-grade sarcomas.

The proportion of patients receiving postoperative RT was highest between 2008–2014 (42%) and slightly decreased thereafter (36%). Use of preoperative RT increased over time, from 1.2% in the earliest period to 8.1% in the most recent period. The proportion of patients treated with RT alone also increased, from 6.6% to 12%. Other treatment modalities, including surgery alone or systemic therapy combinations, remained relatively stable and were used in a minority of patients (see **Table 5**).

**Table 5:** Distribution of treatment strategies for ETS across diagnosis year groups (2000–2007, 2008–2014, 2015–2021).

Characteristic	Overall, N = 2,910*^1^*	2000–2007, N = 334*^1^*	2008–2014, N = 914*^1^*	2015–2021, N = 1,662*^1^*
Treatment				
Postoperative RT	1,065 (37%)	90 (27%)	384 (42%)	591 (36%)
Preoperative RT	182 (6.3%)	4 (1.2%)	44 (4.8%)	134 (8.1%)
RT alone	294 (10%)	22 (6.6%)	68 (7.4%)	204 (12%)
Surgery alone	131 (4.5%)	18 (5.4%)	47 (5.1%)	66 (4.0%)
Systemic Therapy + RT	6 (0.2%)	3 (0.9%)	3 (0.3%)	0 (0%)
Systemic Therapy alone	113 (3.9%)	19 (5.7%)	30 (3.3%)	64 (3.9%)
Unknown	1,119 (38%)	178 (53%)	338 (37%)	603 (36%)

*^1^* n (%).

### Sensitivity analysis

To assess the robustness of our multivariable Cox regression results in the presence of missing data, we conducted a sensitivity analysis using multiple imputation with five imputations. The imputed model results were compared to a complete-case analysis based on the non-imputed data (referred to as “real” data).

Overall, the effect estimates from the imputed model were consistent with those obtained from the complete-case analysis, supporting the validity of the results. While some differences in standard errors and p-values were observed - particularly for sex and histological grade - the direction and magnitude of associations remained stable. Notably, higher age and advanced T-status were significantly associated with increased mortality risk in both models. Treatment with RT alone was also associated with worse survival compared to adjuvant RT across both approaches.

A detailed tabular comparison of both models is presented in **Supplementary Table S3**.

## Discussion

The specific aim of this study was to evaluate treatment strategies and therapeutic outcomes of extremity sarcoma patients based on German cancer registry data. We compared our findings to the German S3 guideline and the National Comprehensive Cancer Network^®^ (NCCN^®^) guideline recommendations (1) as benchmark.

In our analysis, data quality was limited by the incompleteness of TNM-stages. T-stages could not be assessed in 26% of STS cases. N-stage data were missing in 28% of collected cases, while information on distant metastasis was missing in 27% of STS cases (see **Supplementary Table S1**). Likewise, clinical performance status was reported only by two of the three included cancer registries. An important prognostic factor, information on clinical performance should always be submitted to the cancer registries. Therefore, data transfers from oncological centers should be obtained with a higher degree of data completeness to improve data quality. A publication by the European Network of Cancer Registries stated that only about 50% of European cancer registries collect additional clinical data, but this is limited data quality especially with respect to treatment strategies, such as surgery or chemotherapy yes/no (33). Unfortunately, only a few cancer registries collect clinical data to assess the quality of care, due to federal regulations (34).

Katalinic et al. estimated that based on organ German cancer center certification data, that only 50% of all cancer patients in Germany are treated in annually certified cancer centers. Therefore, treatment quality of all cancer patients in Germany is still relatively unknown (35). Another German study showed a high accordance of recommendations of the tumor board with the guideline recommendations. In this analysis, over 90% of recommendations were either adherent with guidelines (75.5%) or over fulfilling guideline recommendations (15.6%) (36). This study indicates that treatment quality within oncological centers is high. Moreover, the implementation of sarcoma registries could help to improve data quality in analogy to already existing entity-specific registries (37).

For early-stage STS, both guidelines recommend surgical wide resection with an intent to obtain negative margins (7, 38). Low-grade sarcomas of all T-stages (T1-T4, N0, M0, low-grade) with surgical margins less than 1cm and without intact fascial plane should be considered for re-resection (39). The NCCN guideline recommends the use of RT as an adjunct to surgery as supported by prospective studies based on an improvement in disease-free survival (DFS) although not OS (9, 10, 12).

Treatment options for high-grade sarcoma patients should be determined by an experienced multidisciplinary team based on patient related factors such as the patient´s age, performance status, comorbidities, and location and histological subtype of the tumor (1). In our multivariable Cox regression, higher age, high tumor grade, and advanced T-status were significantly associated with increased mortality. According to the German S3-guideline recommendations, pre- and postoperative RT in STS of the extremities and trunk is associated with better OS, while preoperative RT lowers local recurrence rates as effective as postoperative RT with lower radiation doses (18, 40). Moreover, neoadjuvant RT was associated with significantly better OS compared to adjuvant RT (18). In general, the NCCN guideline recommends surgery preceded or followed by RT for patients with stage II tumors (T1, N0, M0, G2-3) that are resectable with acceptable functional outcomes (17, 18). According to these recommendations, adjuvant RT and surgery alone were associated with the most favorable survival outcomes in patients with ETS in our data.

In contrast to the guidelines, preoperative RT was not associated with a survival benefit for ETS patients compared to postoperative RT (postoperative RT vs. preoperative RT HR: 1.04, 95%CI 0.49–2.23). This lack of observed benefit for neoadjuvant RT should, however, be interpreted with caution due to the small number of patients who received preoperative RT (n = 139), which substantially limits the statistical power of this comparison. The resulting wide confidence interval underscores the imprecision of the effect estimate and increases the risk of a type II error. Thus, the absence of a statistically significant advantage in our study does not necessarily imply equivalence or inferiority of preoperative RT. Larger studies or meta-analyses are needed to more definitively assess the comparative survival outcomes of neoadjuvant versus adjuvant RT in a real-world setting.

Moreover, we acknowledge that selection bias in assigning patients to preoperative versus postoperative RT is an important concern in this type of registry-based analysis. In our cohort, only approximately 10% of patients received preoperative RT, reflecting common practice patterns in German sarcoma centers. This disproportion likely reflects institutional preferences, logistical considerations, or patient-related factors, and not random assignment. As such, treatment sequencing may be confounded by unmeasured clinical variables. While PSM was used to adjust for known covariates, this approach cannot fully account for unmeasured confounders such as resectability, multidisciplinary decision-making processes, or tumor proximity to critical structures.

The findings from our cohort demonstrate clear temporal shifts in treatment sequencing over the study period, which may represent an important confounding factor when interpreting survival outcomes. As shown in **Table 5**, the use of preoperative RT increased steadily from 1.2% in 2000–2007 to 8.1% in 2015–2021. This trend aligns with international developments, particularly following the publication of the landmark trial by O’Sullivan et al. in 2002, which supported the oncologic equivalence of preoperative RT with reduced long-term toxicity (18). However, it likely took several years for these findings to be translated into clinical practice, especially outside of academic centers.

Simultaneously, we observed a decline in the proportion of cases with unknown treatment information over time (from 53% to 36%), which may reflect improvements in documentation and registry quality. Interestingly, the proportion of patients receiving postoperative RT remained relatively stable but peaked between 2008–2014, suggesting ongoing reliance on postoperative sequencing during the early period of preoperative RT adoption. These shifting treatment patterns over time reflect broader changes in clinical guidelines, institutional practices, and registry completeness, and likely introduce time-dependent confounding into observational analyses. Although we adjusted for year of diagnosis and performed stratified analyses, such secular trends cannot be fully eliminated in retrospective studies and must be considered when interpreting comparative treatment outcomes.

Therefore, the non-randomized treatment assignment and limited representation of preoperative RT must be considered a potential limitation of our analysis, and our findings should be interpreted in that context. To evaluate the prognostic value of preoperative RT for sarcoma patients in a real-world-setting, larger cohorts of patients treated with preoperative RT are needed.

A recent population-based analysis by Dunlop et al. (2024) investigated the impact of neighborhood socioeconomic status (nSES) on treatment adherence and outcomes in patients with high-grade, large extremity soft tissue sarcomas within the SEER registry. Their study revealed that patients from lower nSES areas were significantly less likely to receive RT in accordance with NCCN Guideline recommendations and demonstrated poorer cancer-specific survival (HR 1.19, 95% CI 1.01–1.40). Notably, 21.3% of their cohort did not receive RT at all, despite fulfilling criteria for guideline-concordant treatment. These findings underscore the critical role of structural and socioeconomic factors in access to optimal multimodal sarcoma therapy (41). While our analysis did not directly examine nSES, a considerable proportion of patients in our cohort (43%) also lacked documented RT, pointing toward potential underreporting or undertreatment in the real-world setting.

Taken together, both datasets suggest that even in high-income healthcare systems, there may be relevant barriers—whether organizational, geographic, or socioeconomic—that affect the implementation of evidence-based care in sarcoma patients. This highlights the urgent need for health system-level strategies to identify at-risk populations, ensure equitable access to multimodal therapy, and enhance compliance with established treatment guidelines.

Surgery alone could be considered for small high-grade tumors with wider surgical margins (1). Furthermore, NCCN states that surgery followed by RT is the primary treatment for patients with stage IIIA or IIIB tumors that are resectable with acceptable functionality (1). Our findings are in line with those reported by Matsuoka et al. (2025), who conducted a SEER-based analysis focusing specifically on non-operative patients with localized extremity non-small round cell sarcomas. In their study, RT was associated with significantly improved cancer-specific survival (CSS) and OS, despite the overall poor prognosis in this high-risk cohort. Notably, their reported 5-year OS rates ranged from 14% in AJCC stage IIIB to 56% in stage IB, and RT was more frequently administered in patients with advanced disease stages (42). In contrast, our analysis focused on a broader cohort of patients with extremity sarcomas, including both operative and non-operative cases, within a real-world German cancer registry. Importantly, we observed that RT alone was associated with significantly worse survival compared to surgery plus adjuvant RT, and even compared to surgery alone. This difference likely reflects both the inferior prognosis of patients not eligible for surgery and the curative potential of resection, which remains the cornerstone of sarcoma therapy. Our results underscore that RT cannot fully compensate for the absence of surgery, even though it may provide benefit in selected non-operative cases, as highlighted by Matsuoka et al.

We would like to highlight that the benefits of RT based on the randomized data are local control, not OS: However, information on tumor progression and local control are limited in cancer registries. Therefore, we focused on OS as primary endpoint. The absence of an OS benefit in some of their subgroups does not necessarily indicate that RT should not be considered in a multimodal therapeutic approach.

### Implications for clinical decision-making

Taken together, our findings provide important real-world insights that may support clinical decision-making in patients with extremity soft tissue sarcomas. While guideline-adherent use of surgery and adjuvant RT remains the most evidence-based and commonly used strategy, our results suggest that preoperative RT—although supported by prospective data—has not yet been widely adopted in Germany and was not associated with improved OS in this analysis. Given the limitations of our data, including small sample size for preoperative RT and potential selection biases, this finding should not discourage its use but rather emphasize the need for individualized treatment planning.

In practice, the choice between preoperative and postoperative RT should continue to be made in multidisciplinary tumor boards, weighing the potential benefits in terms of local control and toxicity against surgical feasibility and patient-specific factors. Our findings reinforce the importance of tailoring treatment strategies to tumor biology (e.g., grade, size, resectability) and patient-related factors (e.g., comorbidities, age, functional status). In patients with high-grade, resectable ETS, the combination of surgery and RT remains the standard of care, with surgery alone potentially being reserved for small tumors with clear margins and favorable histology.

Importantly, the observed underuse or underreporting of RT in nearly half of all cases underlines a potential gap in guideline adherence and raises awareness for the need to improve both clinical documentation and access to multimodal therapy. For clinicians, this finding highlights the importance of reviewing each case for eligibility for RT—even in settings where surgical margins appear satisfactory. Additionally, more widespread implementation of standardized sarcoma documentation tools, registry enhancements, and national quality assurance initiatives may help ensure that all eligible patients receive the full benefit of evidence-based care.

In summary, our data support current guideline-based treatment pathways but also reveal real-world limitations and practice variations that must be considered during shared decision-making. By identifying structural and clinical factors that influence treatment delivery and outcome, our study encourages a more nuanced, individualized, and guideline-conscious approach to the management of ETS.

## Conclusions

Our findings highlight the prognostic relevance of tumor grade, T-stage, and patient age in the survival of extremity sarcoma patients. Among treatment strategies, surgery alone and adjuvant RT were associated with the most favorable survival outcomes, whereas RT without surgery was linked to significantly poorer prognosis. These results support current clinical guidelines emphasizing multimodal therapy approaches, particularly the role of surgery with curative intent.

Given the limitations of registry-based retrospective analyses, including missing data and potential residual confounding, further prospective studies are needed to clarify the optimal sequencing and combination of treatments. Future research should also explore patient-centered outcomes such as quality of life and functional status, and assess the potential of novel technologies (e.g., AI-based risk stratification) to support individualized treatment decisions in sarcoma care.

## Data Availability

The raw data supporting the conclusions of this article will be made available by the authors, without undue reservation.

## Supplementary material

Supplementary Table 1 Patients characteristics of all included extremity sarcoma patients. ECOG, Eastern Cooperative Oncology Group.

Supplementary Table 2 Numeric results of propensity score matching.

Supplementary Table 3 Comparison of multivariable Cox regression models using multiple imputation (pooled estimates) and complete-case analysis (real data).
