# Supplementary material for: Treatment strategies for extremity sarcoma patients: a population-based analysis on German clinical cancer registry data
Source: Front Oncol. 2025 May 29;15:1555502. doi: 10.3389/fonc.2025.1555502 (PMC12179432; doi:10.3389/fonc.2025.1555502)
Supplement: Supplementary Table 1 — Patients characteristics of all included extremity sarcoma patients. ECOG, Eastern Cooperative Oncology Group. [file Table1.docx]

| **Characteristic** | **Median Survival (95% CI)** | **p-value***^1^* | |
| --- | --- | --- | --- |
| Age |  | 0.023 |  |
| <75 | 22 (10, 35) |  |  |
| ≥75 | 36 (31, 65) |  |  |
| Sex |  | 0.7 |  |
| female | 35 (20, 94) |  |  |
| male | 27 (20, 38) |  |  |
| Histologie |  | 0.7 |  |
| adenocarcinoma | 31 (9.0, 65) |  |  |
| large cell lung carcinoma | 35 (—, —) |  |  |
| NOS | 46 (36, —) |  |  |
| squamous cell carcinoma | 27 (24, 65) |  |  |
| Grading |  | 0.2 |  |
| known | 38 (32, 94) |  |  |
| unknown | 26 (18, 32) |  |  |
| CCI |  | >0.9 |  |
| <3 | 28 (—, —) |  |  |
| >8 | 34 (11, —) |  |  |
| 3-5 | 32 (24, —) |  |  |
| 6-8 | 26 (18, 42) |  |  |
| Karnofsky-Index (%) |  | 0.3 |  |
| >70 | 35 (24, 65) |  |  |
| ≤70 | 27 (18, 38) |  |  |
| PET |  | 0.8 |  |
| no | 29 (10, 94) |  |  |
| yes | 31 (21, 42) |  |  |

Table S1: Median Survival rates for different subgroups
