# Supplementary material for: Treatment strategies for extremity sarcoma patients: a population-based analysis on German clinical cancer registry data
Source: Front Oncol. 2025 May 29;15:1555502. doi: 10.3389/fonc.2025.1555502 (PMC12179432; doi:10.3389/fonc.2025.1555502)
Supplement: Supplementary file 2 [file DataSheet1.docx]

# Supplement

| **Characteristic** | **N = 2,689***^1^* |
| --- | --- |
| **Sex** |  |
| Male | 1,523 (57%) |
| Female | 1,166 (43%) |
| **Age** (years) | 65 (52, 76) |
| **Histological subtype** |  |
| Clear Cell Sarcoma | 31 (1.2%) |
| Dermatofibrosarcoma Protuberans | 17 (0.6%) |
| Epithelioid Sarcoma | 26 (1.0%) |
| Fibromyxoid Sarcoma | 57 (2.1%) |
| Fibrosarcoma | 65 (2.4%) |
| Leiomyosarcoma | 384 (14%) |
| Liposarcoma | 536 (20%) |
| Malignant Fibrous Histiocytoma | 42 (1.6%) |
| Malignant Peripheral Nerve Sheath Tumor | 21 (0.8%) |
| Malignant Solitary Fibrous Tumor | 2 (<0.1%) |
| Myxofibrosarcoma | 296 (11%) |
| Myxosarcoma | 21 (0.8%) |
| Pleomorphic Sarcoma | 256 (9.5%) |
| Unclassified Soft Tissue Sarcoma | 644 (24%) |
| Spindle Cell Sarcoma | 95 (3.5%) |
| Synovial Sarcoma | 131 (4.9%) |
| Undifferentiated Sarcoma | 65 (2.4%) |
| **Histological grade** |  |
| High Grade | 982 (37%) |
| Low Grade | 1,040 (39%) |
| Unknown | 667 (25%) |
| **T-status** |  |
| 1 | 515 (19%) |
| 2 | 966 (36%) |
| 3 | 159 (5.9%) |
| 4 | 174 (6.5%) |
| Unknown | 700 (26%) |
| X | 175 (6.5%) |
| **N-status** |  |
| 0 | 1,630 (61%) |
| 1 | 50 (1.9%) |
| Unknown | 748 (28%) |
| X | 261 (9.7%) |
| **M-Status** |  |
| 0 | 1,669 (62%) |
| 1 | 224 (8.3%) |
| Unknown | 737 (27%) |
| X | 59 (2.2%) |
| **ECOG-status** |  |
| 0 | 247 (9.2%) |
| 1 | 173 (6.4%) |
| 2 | 69 (2.6%) |
| 3 | 21 (0.8%) |
| 4 | 5 (0.2%) |
| Unknown | 2,174 (81%) |
| **Treatment** |  |
| Adjuvant RT | 998 (37%) |
| Neoadjuvant RT | 175 (6.5%) |
| RT alone | 265 (9.9%) |
| Surgery alone | 122 (4.5%) |
| Systemic Therapy + RT | 6 (0.2%) |
| Systemic Therapy alone | 96 (3.6%) |
| Unknown | 1,027 (38%) |
| **Tumor localization** |  |
| Lower Extremity | 2,014 (75%) |
| Upper Extremity | 675 (25%) |
| **Tumor side** |  |
| Both Sides | 1 (<0.1%) |
| Left Side | 873 (32%) |
| Midline | 1 (<0.1%) |
| Right Side | 874 (33%) |
| Unknown | 940 (35%) |
| **Tumor size** |  |
| <=5cm | 515 (19%) |
| >10cm and <15cm | 159 (5.9%) |
| >15cm | 174 (6.5%) |
| >5cm and <10cm | 966 (36%) |
| Unknown | 875 (33%) |
| **Resection status** |  |
| R0-Status | 1,263 (67%) |
| R1-Status | 281 (15%) |
| R2-Status | 49 (2.6%) |
| RX-Status | 282 (15%) |
| Unknown | 814 |
| *^1^* n (%); Median (Q1, Q3) | |

Table S1 Patients characteristics of all included extremity sarcoma patients. ECOG = Eastern Cooperative Oncology Group.

Call:

matchit(formula = Radiatio ~ alter_ge + SEX + HISTGRAD_einfach +

T_Status + ECOG_Status, data = data, method = "nearest",

ratio = 1)

Summary of Balance for All Data:

Means Treated Means Control Std. Mean Diff.

distance 0.6039 0.5549 0.4639

alter_ge 61.6934 63.9446 -0.1390

SEXM 0.5711 0.5598 0.0227

SEXW 0.4289 0.4402 -0.0227

HISTGRAD_einfachHigh Grade 0.4283 0.2768 0.3062

HISTGRAD_einfachLow Grade 0.3525 0.4348 -0.1724

HISTGRAD_einfachUnknown 0.2192 0.2884 -0.1671

T_Status1 0.1797 0.2080 -0.0737

T_Status2 0.4073 0.2920 0.2347

T_Status3 0.0612 0.0562 0.0206

T_Status4 0.0707 0.0562 0.0565

T_StatusUnknown 0.2250 0.3098 -0.2032

T_StatusX 0.0561 0.0777 -0.0938

ECOG_Status0 0.0911 0.0929 -0.0060

ECOG_Status1 0.0727 0.0527 0.0770

ECOG_Status2 0.0274 0.0232 0.0257

ECOG_Status3 0.0051 0.0116 -0.0914

ECOG_Status4 0.0006 0.0036 -0.1163

ECOG_StatusUnknown 0.8031 0.8161 -0.0327

Var. Ratio eCDF Mean eCDF Max

distance 0.9769 0.1321 0.2171

alter_ge 0.8380 0.0311 0.0799

SEXM . 0.0112 0.0112

SEXW . 0.0112 0.0112

HISTGRAD_einfachHigh Grade . 0.1515 0.1515

HISTGRAD_einfachLow Grade . 0.0824 0.0824

HISTGRAD_einfachUnknown . 0.0691 0.0691

T_Status1 . 0.0283 0.0283

T_Status2 . 0.1153 0.1153

T_Status3 . 0.0049 0.0049

T_Status4 . 0.0145 0.0145

T_StatusUnknown . 0.0848 0.0848

T_StatusX . 0.0216 0.0216

ECOG_Status0 . 0.0017 0.0017

ECOG_Status1 . 0.0200 0.0200

ECOG_Status2 . 0.0042 0.0042

ECOG_Status3 . 0.0065 0.0065

ECOG_Status4 . 0.0029 0.0029

ECOG_StatusUnknown . 0.0130 0.0130

Summary of Balance for Matched Data:

Means Treated Means Control Std. Mean Diff.

distance 0.6568 0.5549 0.9653

alter_ge 59.9062 63.9446 -0.2493

SEXM 0.5696 0.5598 0.0198

SEXW 0.4304 0.4402 -0.0198

HISTGRAD_einfachHigh Grade 0.5964 0.2768 0.6460

HISTGRAD_einfachLow Grade 0.2741 0.4348 -0.3364

HISTGRAD_einfachUnknown 0.1295 0.2884 -0.3841

T_Status1 0.1366 0.2080 -0.1860

T_Status2 0.5536 0.2920 0.5325

T_Status3 0.0652 0.0562 0.0373

T_Status4 0.0866 0.0562 0.1184

T_StatusUnknown 0.1295 0.3098 -0.4319

T_StatusX 0.0286 0.0777 -0.2134

ECOG_Status0 0.0955 0.0929 0.0093

ECOG_Status1 0.0884 0.0527 0.1376

ECOG_Status2 0.0304 0.0232 0.0438

ECOG_Status3 0.0000 0.0116 -0.1630

ECOG_Status4 0.0000 0.0036 -0.1415

ECOG_StatusUnknown 0.7857 0.8161 -0.0763

Var. Ratio eCDF Mean eCDF Max Std. Pair Dist.

distance 0.4391 0.2809 0.4768 0.9654

alter_ge 0.9122 0.0489 0.1098 1.2182

SEXM . 0.0098 0.0098 0.9688

SEXW . 0.0098 0.0098 0.9688

HISTGRAD_einfachHigh Grade . 0.3196 0.3196 0.7614

HISTGRAD_einfachLow Grade . 0.1607 0.1607 0.8485

HISTGRAD_einfachUnknown . 0.1589 0.1589 0.7510

T_Status1 . 0.0714 0.0714 0.7488

T_Status2 . 0.2616 0.2616 0.9213

T_Status3 . 0.0089 0.0089 0.4545

T_Status4 . 0.0304 0.0304 0.5223

T_StatusUnknown . 0.1804 0.1804 0.7655

T_StatusX . 0.0491 0.0491 0.4463

ECOG_Status0 . 0.0027 0.0027 0.5925

ECOG_Status1 . 0.0357 0.0357 0.4953

ECOG_Status2 . 0.0071 0.0071 0.3063

ECOG_Status3 . 0.0116 0.0116 0.1630

ECOG_Status4 . 0.0036 0.0036 0.1415

ECOG_StatusUnknown . 0.0304 0.0304 0.7813

Sample Sizes:

Control Treated

All 1120 1569

Matched 1120 1120

Unmatched 0 449

Discarded 0 0

Table S2 numeric results of propensity score matching

| **Characteristic** | **Estimate (Pooled)** | **Std. Error (Pooled)** | **P-value (Pooled)** | **Estimate (Real)** | **Std. Error (Real)** | **P-value (Real)** |
| --- | --- | --- | --- | --- | --- | --- |
| SEXW | -0.28 | 0.13 | 0.0346 | -0.25 | 0.16 | 0.106 |
| age_group >80 | 1.66 | 0.24 | < 0.001 | 1.72 | 0.31 | < 0.001 |
| age_group 51-60 | 0.27 | 0.28 | 0.3335 | 0.43 | 0.34 | 0.208 |
| age_group 61-70 | 0.78 | 0.24 | 0.0011 | 0.80 | 0.28 | 0.0043 |
| age_group 71-80 | 1.18 | 0.23 | < 0.001 | 1.19 | 0.30 | < 0.001 |
| Tumor_localisation Upper Extremity | -0.05 | 0.16 | 0.7495 | 0.27 | 0.19 | 0.156 |
| HISTGRAD_ Low Grade | -0.54 | 0.15 | < 0.001 | -0.46 | 0.21 | 0.0316 |
| HISTGRAD_ Unknown | -0.50 | 0.17 | 0.0029 | -0.66 | 0.21 | 0.0019 |
| T_Status 2 | -0.05 | 0.22 | 0.8043 | -0.24 | 0.26 | 0.355 |
| T_Status 3 | 1.83 | 0.40 | < 0.001 | 1.66 | 0.50 | 0.00091 |
| T_Status 4 | 2.08 | 0.34 | < 0.001 | 1.90 | 0.41 | < 0.001 |
| T_Status Unknown | -0.21 | 0.23 | 0.3493 | -0.38 | 0.28 | 0.1745 |
| T_Status X | 0.24 | 0.28 | 0.3856 | 0.29 | 0.37 | 0.4278 |
| Treatment Neoadjuvant RT | 0.13 | 0.32 | 0.6934 | 0.08 | 0.39 | 0.838 |
| Treatment RT alone | 0.59 | 0.16 | < 0.001 | 0.56 | 0.24 | 0.0222 |
| Treatment Surgery alone | 0.11 | 0.19 | 0.5585 | 0.03 | 0.23 | 0.880 |

Table S3 Comparison of multivariable Cox regression models using multiple imputation (pooled estimates) and complete-case analysis (real data)
